# Access to healthcare for people with intellectual disability

**DOI:** 10.1186/1472-6955-14-S1-S1

**Published:** 2015-10-08

**Authors:** Magnus Sandberg, Gerd Ahlström, Jimmie Kristensson

**Affiliations:** 1 Department of Health Sciences, Faculty of Medicine, Lund University, P.O. Box 157, SE-221 00 Lund, Sweden

## Background

WHO has expressed that people with intellectual disabilities (ID) often are disadvantaged when it comes to accessing healthcare. However, a more solid evidence base is needed to determine if, and in which parts of the health system people with ID have lower access.

## Aim

To explore somatic healthcare utilisation patterns among people with ID, 55 years and older, in comparison with the general population in Sweden 2002-2012.

## Methods

This was a longitudinal, retrospective, population register-based, case-control study. The ID group included 7,936 people aged 55+ who in 2012 received services according to the Act concerning Support and Service for Persons with Certain Functional Impairments, and who were classified with ID from birth or early age. An equally large matched control group was selected from the Swedish Population Register. The sample was divided into four age groups; 55-59, 60–63, 64–69; and >69 years. In- and outpatient data was collected from the Swedish National Patient Register for 2002-2012.

## Results

In the three youngest age groups people in the ID group had in general higher rates of acute in- and outpatient care and longer acute length of stay. For planned in- and outpatient care few significant differences were seen in the three youngest age groups, but with higher rates in the control group for the oldest age group.

## Conclusions

Despite high acute care, the health system seems to be unable to give people with ID access to planned health care. One reason may be inadequate competence among healthcare professionals. However, this needs to be investigated more deeply before any complex intervention with the aim to increase access to planned healthcare is developed.

